# A Giant Euthyroid Endemic Multinodular Goiter with No Obstructive or Compressive Symptoms

**DOI:** 10.1155/2011/620480

**Published:** 2011-06-13

**Authors:** Ahmed Nada, Ashraf Mohamed Ahmed, Ramon Vilallonga, Manuel Armengol, Ibrahim Moustafa

**Affiliations:** ^1^General Surgery Department, Cairo University, 28 Mourad street, Giza, Egypt; ^2^General Surgery Department, Cairo-Fataemic Hospital, 85 Manial St., Cairo 11451, Egypt; ^3^General Surgery Department, University Hospital Vall d'Hebron, 08035, Barcelona, Spain

## Abstract

Diffusely enlarged thyroid glands (goitres) are becoming increasingly infrequent. However, in some geographical areas they are still relatively common and can cause compressive symptoms involving the trachea, oesophagus, and recurrent laryngeal nerve. Surgical treatment of diffusely enlarged thyroid glands requires a high level of expertise and may lead to severe complications. Here we present a case report of surgical treatment of an extremely enlarged thyroid gland, found in a 61-year-old female patient. The patient underwent surgery, and a thyroidectomy was performed. The resulting specimen weighed 4.7 kg (10.4 lbs). Histopathological examination revealed a multinodular goitre with multiple cysts and areas of haemorrhage and necrosis. Surgical excision can immediately resolve local symptoms and is often recommended when substernal extension is evident. To the best of our knowledge, this is the largest thyroid gland ever reported in the literature. Only experienced surgeons should treat large thyroid goitres. Ideally, large thyroid goitres should be treated before they reach a substernal component, otherwise any sudden growth in gland size could seriously compromise respiration.

## 1. Introduction

Goitres (from the Latin *guttur*, throat), which are defined as an enlargement of the thyroid, have been a recognized medical condition since 2700 B.C., even though the thyroid gland itself was not documented until the Renaissance period. In 1619, Hieronymus Fabricius ab Aquapendente recognized that goitres arise from the thyroid gland. The actual term thyroid gland (Greek *thyreoeides*, shield-shaped) is, however, attributed to Thomas Wharton in *Adenographia* (1656) [1]. Diffusely enlarged thyroid glands can cause compressive symptoms involving the trachea, oesophagus, and recurrent laryngeal nerve. These symptoms are usually associated with malignant goitres, and benign nodular goitres do not normally cause obstructive symptoms [2]. In this paper, we present a case of a giant endemic euthyroid multi-nodular goitre, with no obstructive or compressive symptoms.

## 2. Case Report

**Figure 1:**
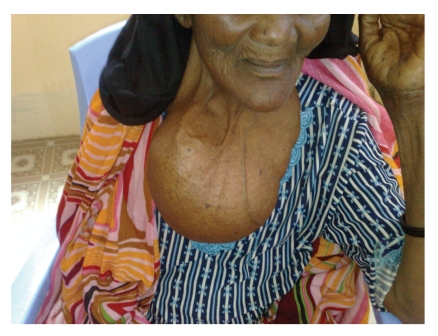
External view of the neck of the patient who underwent to surgery. Observe the large gland.

In a trip to Geneina, Darfur, Sudan organized by the Arab Medical Union, in a voluntary medical mission to a local hospital, our team operated on a 61-year-old female patient (with 11 sons and daughters, the youngest of which was 31 years old). The patient had no concomitant morbidity, no past history (surgical or medical), and no relevant family medical history. The patient presented with a huge neck swelling that was interfering with her normal movements, and which she complained was giving her a sense of tightness and heaviness (Figure 1). Obviously, cosmetic aspects of such a large goitre were also mentioned. The patient first noticed the goitre 40 years ago, which gradually progressed in size, but with no obstructive or compressive symptoms. The goitre measured approximately 27 × 23 cm in its largest dimensions and displayed multidirectional enlargement: anteriorly, where it was pendulous over the sternum; posteriorly; superiorly, to the mandible and floor of the mouth; and inferiorly, where the lower border or edge of the goitre could not be palpated (suggesting substernal extension). The goitre did not display mobility with deglutition. A thyroid hormone profile confirmed the euthyroid status of the patient during routine preoperative tests. Although we considered the possibility of malignancy, no logistic medical facilities were available to aid in diagnosis such as the fine needle aspiration cytology (FNAC). Therefore, the decision to operate was made based on the patient's complaints and the possibility of a hidden malignancy. 

During surgery, contrary to our expectations, we were able to smoothly intubate the patient. We started with a transverse elliptical excision of the redundant skin. The strap muscles were thinned-out and had to be cut for proper exposure. After formal exposure of the thyroid gland, the swelling was observed to extend in all directions: upwards to the base of the tongue; downwards, behind the sternum, down to manubrio-sternal junction; and backwards, lying on the vertebrae. Anatomical landmarks were disturbed to such an extent that carotid sheaths were adhered to the swelling, nearly constituting one mass. Feeding and draining vessels were megalised and engorged. We proceeded with formal total thyroidectomy, starting with the right lobe (the largest lobe). Although we could not identify the recurrent laryngeal nerves (our routine practice), we were able to identify and preserve 2 of the parathyroid glands (on the left side). 

After completion of the thyroid excision, tracheomalacia was observed, which necessitated insertion of a tracheostomy. Before the tracheostomy, a trial of extubation was attempted by the anesthetist, but stridor and lowered arterial O2 saturation occurred. Therefore, we inserted the tracheostomy. Closure of the wound was quite difficult because of the thinned-out strap muscles, nearly atrophied platysma muscle and the redundant skin. 

**Figure 2:**
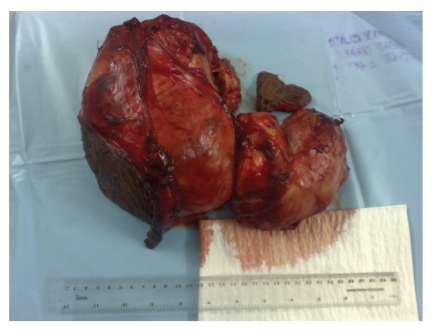
Specimen of the thyroid.

The resulting thyroid specimen was 35 × 27 cm in its largest dimensions and weighed 4.7 kg (Figure 2). Histopathological examination showed a multi-nodular goitre with multiple cysts and areas of haemorrhage and necrosis. The patient was kept under our supervision at a local hospital for 2 days, with no postoperative complications, but had to be transferred to the ICU of a separate hospital, because of lack of personnel, equipment, and other resources at this primitive local hospital. Unfortunately, because of a lack of experience at that hospital, the patient died from a mucous plug in the tracheostomy tube, two days after surgery.

## 3. Discussion

A multi-nodular goitre is simply a thyroid gland that is usually enlarged and contains multiple thyroid nodules. The nodules can be very small, often only a few millimetres in size, or can be larger, perhaps several cm each [2]. The normal adult thyroid gland weighs 10–25 g [3]. The term endemic goitre refers to the occurrence of goitre in a significant proportion of individuals in a particular geographic region [1]. 

Worldwide, the most common cause of goitre is iodine deficiency. In fact, it has been estimated that goitres affect as many as 200 million of the 800 million people who have a diet deficient in iodine [3]. 

Currently, iodine deficiency disorder is associated with the presence of endemic goitre in India, China, Central Asia, and Central Africa. In Sudan, several goitre prevalence surveys conducted by the Government have identified geographical regions at high risk for iodine deficiency disorder, including Darfur State (TGR = 87%, 5885 individuals were examined, including children <18 years of age and female adults <45 years of age) [4]. Endemic goitre is thought to be caused by TSH stimulation resulting from inadequate thyroid hormone and other paracrine growth factor synthesis. Thus, the thyroid gland enlarges in an attempt to maintain a euthyroid state [1]. Substernal goitre is an unusual presentation of an intrathoracic extension of an enlarged thyroid, usually resulting from multi-nodular goitre [5]. Diffusely enlarged thyroid glands can cause compressive symptoms involving the trachea, oesophagus, and recurrent laryngeal nerve. Some of these compressive symptoms can include dyspnea, stridor, orthopnea, dysphagia, or hoarseness. This is particularly concerning when there is a substernal component to these lesions, because any sudden growth in gland size would occur within a confined space and could seriously compromise respiration [6]. However, these specific symptoms can also occur in malignant goitres [2]. Importantly, based on the unfortunate outcome for our patient, one should avoid unnecessary and risky surgeries when there is a lack of facilities for proper postoperative care.

The risk of malignancy in dominant nodules within multi-nodular goitres is approximately 10% [7]. Such malignancies can be extremely slow growing and may be present for many years before being discovered [8]. Surgical excision can immediately resolve local symptoms and is often recommended when substernal extension is evident. Although goitres have been reported to decrease by up to 40% after RAI treatment, such therapy may not sufficiently diminish the size of the gland and could even cause temporary gland enlargement due to subsequent oedema. These enlarged thyroid glands can also be of cosmetic concern for some patients, who opt for surgical excision of the lobe or gland for this reason [6].

## 4. Conclusion

In the 21st century, there are still living examples of some historical pathologies. These afflictions are predominantly found in areas suffering from a lack of primary health care services, as a result of social, economic, and political problems. In the case study presented here, the patient's pathology was entirely preventive in origin. Moreover, although the operation was performed smoothly and successfully, death occurred for essentially the same reasons as her disease. International Health Organizations should focus on these areas of the world to eliminate such ignorance and lack of resources, at least with respect to health issues. Although the surgical technique used was routine; the operative challenge was the magnificent size of the gland and its adherence to vital structures. To improve the outcome of such operations, which are commonly performed worldwide, such huge goitres must be taken seriously. In particular, the establishment of safety measures and proper facilities (both equipment and personnel) should be considered beforehand.

## References

[1] Clark OH, Lal G, Brunicardi FC, Anderson DK, Billiar TR, Dunn DL, Hunter JG, Poliock RE (2007). Thyroid, parathyroid, and adrenal. *Schwartz’s Principles of Surgery*.

[2] Wilson JD, Foster DW (1992). The thyroid gland. *Williams Textbook of Endocrinology*.

[3] Becker KL, Bilezikian JP, Bremner WJ (1995). Nontoxic goiter. *Principles and Practice of Endocrinology and Metabolism*.

[4] Hussein I Private communication to I C C IDD. http://www.iodinenetwork.net/countries/Sudan.html.

[5] Hanks JB, Townsend C-J, Beauchamp RD, Evers BM, Mattox KL (2004). Endocrine-thyroid. *Sabiston Textbook of Surgery*.

[6] Clark OH, Caron NR, Fischer JE (2007). Endocrine surgery-fine needle aspiration biopsy of the thyroid: thyroid lobectomy and subtotal and total thyroidectomy. *Mastery of Surgery*.

[7] Smith D, Leese G, Cuschieri A, Grace PA, Darzi A, Borley N, Rowley DI (2003). Disorders of the thyroid glands. *Clinical Surgery*.

[8] Harrison BJ, Maddox PR, Smith DM, Cuschieri A, Steel RJC, Mossa AR (2002). Disorders of the thyroid gland. *Essential Surgical Practice*.

